# Developments in ureteroscopic stone treatment: Key themes and remaining challenges

**DOI:** 10.3389/fsurg.2022.1050285

**Published:** 2022-10-24

**Authors:** Russell E. N. Becker, William W. Roberts, Michael E. Lipkin, Khurshid R. Ghani

**Affiliations:** ^1^ Division of Endourology, Department of Urology, Michigan Medicine, University of Michigan, Ann Arbor, MI, United States; ^2^ Division of Urology, Department of Surgery, Duke University School of Medicine, Durham, NC, United States

**Keywords:** lithotripsy, ureteroscopy, outcomes, laser, nephrolithiasis

**Editorial on the Research Topic** Developments in ureteroscopic stone treatment: Key themes and remaining challenges By Becker REN, Roberts WW, Lipkin ME, Ghani KR. (2022) Front. Surg. 9: 1050285. doi: 10.3389/fsurg.2022.1050285

**Figure 1:**
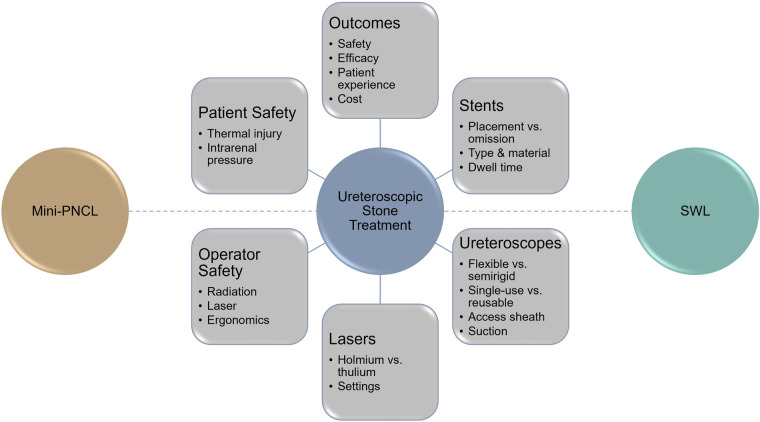
Key themes and remaining challenges in ureteroscopic stone treatment. (Mini-PCNL: mini-percutaneous nephrolithotomy; SWL: shockwave lithotripsy).

The 2022 edition of the Developments in Ureteroscopic Stone Treatment (DUST) symposium brought together an international group of content experts and thought leaders in Miami, Florida for a spirited discussion of recent advances and challenges in the field. The content spanned important themes from preventative management to technological advances in ureteroscopes and working instruments, choices around intraoperative parameters such as laser type, energy settings, irrigation methods, lithotripsy strategies (dusting, fragmenting), stenting practices, and post-operative management. Safety and efficacy outcomes remain paramount, but considerations of cost and the patient experience have also gained considerable recognition and interest. Figure 1 provides a conceptual overview of some of the current themes that are expected to shape discussion over the next decade in the field, several of which are featured in this *Frontiers in Surgery* collection.

One topic of intense interest at the DUST symposium was the proliferation of single-use digital ureteroscopes. As highlighted in work by {Huang et al.} and summarized by {Meng et al.}, these ureteroscopes aim to alleviate some of the challenges of reusable platforms, such as financial cost and complex sterilization. Many new designs also seek to incorporate ergonomic advances such as lighter weight and different grip positions. Single-use platforms have overall become far more affordable, even as their quality has improved to closely rival that of reusable ureteroscopes. Studies comparing safety and efficacy outcomes between single-use and reusable ureteroscopes find they perform similarly overall, and even on parameters such as overall program cost, environmental footprint, and image quality, single-use ureteroscopes are increasingly competitive with reusable platforms.

Similarly, ureteral stent technology continues to improve with the goals of improving patient comfort while maintaining functional performance. {J. Lee et al.} describe several promising new designs in various stages of development. These incorporate a range of innovations from novel stent compositions and coatings to radical reconceptualization of the stent as we know it, such as suture-based stents or dissolvable material.

Other groups continue to push the field forward with novel technologies for active stone fragment evacuation. Removal of fragments and debris from the collecting system during or after laser lithotripsy may theoretically reduce the risk of steinstrasse, stone recurrence, and/or other complications. At the DUST symposium we heard that fluoroscopic-guided steerable aspiration catheters are currently being tested in US clinical trials, while the in-line system described by {Lai et al.}, which maintains synchronous visualization by adapting a rigid ureteral access sheath into a controlled closed-suction fluid system, has been described in China.

A similar controlled fluid system for mini-percutaneous nephrolithotomy (PCNL) has also been developed in China (Endoscopic Surgical Monitoring System, ESMS), which employs inflow and outflow monitoring gauges to facilitate intraoperative calculations of irrigant fluid absorption and blood loss. {Gui et al.} found in a retrospective comparison that patients undergoing mini-PCNL with the ESMS system had significantly reduced irrigant fluid absorption and blood loss, as well as improved postoperative pain scores and return-to-work time, compared to patients undergoing mini-PCNL without ESMS.

A secondary benefit of such regulated fluid systems is the ability to monitor and modulate intrarenal pressures and thermal dissipation. These previously underappreciated intraoperative parameters were heavily emphasized at the DUST symposium, as emerging data continue to elucidate their critical roles in determining key outcomes such as tissue injury, patient pain, and post-procedural infections. As described by {Khusid et al.}, vigilance of intrarenal pressures and the potential for pyelovenous backflow represents an emerging key strategy for preventing infectious complications. Others, which have been the focus of professional society guidelines include preoperative urine testing, evaluation of patient risk factors, and evidence-based antimicrobial prophylaxis.

Recent years have also seen an improved awareness of operator and staff safety, with increased emphasis on provider wellness and career longevity. Alongside our colleagues throughout the health sciences, urologists are increasingly recognizing that we cannot take the best care of our patients, unless we also take care of ourselves. In their review, {Miller & Semins} summarize many of the key considerations for those performing and assisting with ureteroscopy, including radiation safety, laser safety, and surgical ergonomics. The authors provide numerous simple, practical, and easily implemented strategies to help optimize safety for urologists and operating room staff.

As the field continues to work toward optimizing every aspect of the “index” ureteroscopic stone procedure, we have also accumulated better evidence to guide management of special circumstances and subpopulations. The particular challenges of cystinuria are reviewed in detail by {Clark et al.}, including both medical and surgical management. A systematic review and meta-analysis by {Yi et al.} compares surgical options of flexible ureteroscopy and shockwave lithotripsy (SWL) for stone disease in patients with horseshoe kidney, finding that while both are safe with low complication rates, ureteroscopic treatment offers better stone-free rates. Rounding out the collection, {M. Lee et al.} provide an excellent review and practical guideline for workup and management of nephrolithiasis in pregnancy. This is a prime example of how technological and methodological advances in ureteroscopy have led to its adoption as a safe and successful first line treatment option in these complex patients. The evidence-based multidisciplinary consensus statements fill several important gaps in existing society guidelines, and will undoubtedly prove to be an invaluable resource for the on-call general urologist.

DUST 2022 was an expository glimpse into the future of ureteroscopic stone treatment, touching on many exciting new developments, as well as remaining controversies and challenges. For many attendees, the highlight of the meeting once again was the infamous Balloon Debate, which pitted renowned experts against one another on which is the optimal management strategy for an asymptomatic 9 mm lower pole renal stone. Perhaps by DUST 2023 we will be closer to an answer.

